# Physicians’ Investments

**Published:** 1913-07

**Authors:** 


### Physicians' Investments.

We often hear of being let in on the ground floor. A recent advertisement of stock in a diamond mine gives its address as "The Third Floor Annex"—no name of building added. That is about as near the ground floor as doctors ever get in stock investments.

